# Polybrominated Diphenyl Ether (PBDE) Levels in an Expanded Market Basket Survey of U.S. Food and Estimated PBDE Dietary Intake by Age and Sex

**DOI:** 10.1289/ehp.9121

**Published:** 2006-07-13

**Authors:** Arnold Schecter, Olaf Päpke, T. Robert Harris, K.C. Tung, Alice Musumba, James Olson, Linda Birnbaum

**Affiliations:** 1 University of Texas School of Public Health at Southwestern Medical Center, Dallas, Texas, USA; 2 Eurofins-ERGO, Hamburg, Germany; 3 State University of New York Medical Center at Buffalo, Buffalo, New York, USA; 4 National Health and Environmental Effects Research Laboratory, U.S. Environmental Protection Agency, Research Triangle Park, North Carolina USA

**Keywords:** age, dietary intake, market basket survey, PBDEs, polybrominated diphenyl ethers, sex

## Abstract

**Objectives:**

Our objectives in this study were to expand a previously reported U.S. market basket survey using a larger sample size and to estimate levels of PBDE intake from food for the U.S. general population by sex and age.

**Methods:**

We measured concentrations of 13 polybrominated diphenyl ether (PBDE) congeners in food in 62 food samples. In addition, we estimated levels of PBDE intake from food for the U.S. general population by age (birth through ≥60 years of age) and sex.

**Results:**

In food samples, concentrations of total PBDEs varied from 7.9 pg/g (parts per trillion) in milk to 3,726 pg/g in canned sardines. Fish were highest in PBDEs (mean, 1,120 pg/g; median, 616 pg/g; range, 11.14–3,726 pg/g). This was followed by meat (mean, 383 pg/g; median, 190 pg/g; range, 39–1,426 pg/g) and dairy products (mean, 116 pg/g; median, 32.2 pg/g; range, 7.9–683 pg/g). However, using estimates for food consumption (excluding nursing infants), meat accounted for the highest U.S. dietary PBDE intake, followed by dairy and fish, with almost equal contributions. Adult females had lower dietary intake of PBDEs than did adult males, based on body weight. We estimated PBDE intake from food to be 307 ng/kg/day for nursing infants and from 2 ng/kg/day at 2–5 years of age for both males and females to 0.9 ng/kg/day in adult females.

**Conclusion:**

Dietary exposure alone does not appear to account for the very high body burdens measured. The indoor environment (dust, air) may play an important role in PBDE body burdens in addition to food.

Polybrominated diphenyl ethers (PBDEs), persistent and bioaccumulative flame retardants, are of concern because they are ubiquitous in the United States, are potentially toxic, and have been found at rapidly rising levels in humans during the past few decades (Birnbaum and Staskal 2004; Hites 2004; Schecter et al. 2005b; Sjödin et al. 2004; Webster et al. 2005). The high level of PBDE contamination in the U.S. population and food is cause for concern because these compounds are chemically similar to polychlorinated biphenyls (PCBs) and have been shown in laboratory animal studies to be toxic in a number of ways. These include cancer in high-dose studies [National Toxicology Program (NTP) 1986], reproductive and developmental toxicity (Stoker et al. 2004), endocrine disruption (Hallgren and Darnerud 2002), and central nervous system effects (Eriksson et al. 2002; Viberg et al. 2003). PBDEs can be found in some textiles, electronics, (e.g., computers, televisions), plastics, and furniture such as sofas, chairs, and mattresses. Unlike dioxins and PCBs, these chemicals are primarily indoor pollutants and are found at high levels in household vacuum dust and other home and workplace environmental samples (Schecter et al. 2005a; Stapleton et al. 2005).

Very high levels of PBDEs have recently been found in the United States in mothers’ milk (Schecter et al. 2003, 2005b), blood (Mazdai et al. 2003; Morland et al. 2005; Schecter et al. 2004, 2005b; Sjödin et al. 2004), food (Schecter et al. 2004), and adipose tissue (Johnson-Restrepo et al. 2005; She et al. 2002). U.S. blood and milk concentrations were 10- to 20-fold higher than the levels found in Europe (Bocio et al. 2003; Meironyte et al. 1999; Norén and Meironyté 2000; Ohta et al. 2002). Although levels of dioxins, dibenzofurans, and PCBs in human tissues are declining, PBDEs have been increasing substantially in blood levels in the United States during the past two to three decades (Schecter et al. 2005b; Sjödin et al. 2004).

The penta-BDE and octa-BDE commercial PBDE mixtures are no longer being produced or sold in the United States, whereas deca-BDE continues to be manufactured and sold in the United States as well as worldwide. Furthermore, because these compounds are persistent in the environment, reservoir sources are likely to be present for substantial periods of time. These reservoir sources may continue to contaminate food and dust, both of which are believed to contribute substantially to human intake of these compounds (Birnbaum and Staskal 2004; Jones-Otazo et al. 2005; Webster et al. 2005).

Even though studies have begun to estimate PBDE intake from ingestion and inhalation, the amount and percent of intake from food in the U.S. general population have not been well characterized nor have the amounts of intake from dust ingestion and inhalation been well defined (Jones-Otazo et al. 2005; Stapleton et al. 2005; Webster et al. 2005). The present study expands and complements our previous U.S. market basket survey (Schecter et al. 2004) and also estimates dietary PBDE exposure by age and sex from birth through ≥60 years of age. The food sample size is approximately twice the size of the earlier study and includes previously unpublished congener data from that study (Schecter et al. 2004). For the first time, we characterize the U.S. population’s PBDE intake from food.

## Methods

Food samples were purchased during 2003 and 2004 in Dallas, Texas, from three large supermarkets representing national chains. We chose commonly eaten food types and purchased national or store brands whenever possible. Items were frozen and shipped on dry ice to Eurofins-ERGO Laboratory for analysis. The laboratory measured 13 PBDE congeners (BDE-17, BDE-28, BDE-47, BDE-66, BDE-77, BDE-85, BDE-99, BDE-100, BDE-138, BDE-153, BDE-154, BDE-183, and BDE-209) in 62 food samples by gas chromatography-isotope dilution high resolution mass spectrometry. For quality control purposes, one laboratory blank and a quality control pool for each block of samples were run. Quantification was performed only if the sample level was at least twice the blank level. For the analysis of samples of major food types such as fish, meat, cooked egg, cheese, ice cream, and sausage, a total of 5–200 g of the sample was homogenized and mixed with sodium sulfate. Before column extraction, a mixture of internal ^13^C-labeled standards was added to each sample. A mixture of cyclohexane and dichloromethane was applied during column extraction of lipids. After solvent evaporation, gravimetric lipid determination was performed. Fish oil and human milk were used as quality control pools. Details of the analytical procedure have been described elsewhere (Päpke et al. 2004; Schecter et al. 2004). In the present study, we used half the limit of detection (LOD) to estimate levels of congeners below the LOD, whereas we previously calculated them as equal to zero (Schecter et al. 2004).

We used the mean PBDE concentrations in our food samples, in combination with other food consumption estimates, to estimate PBDE intake for several age and sex groups in the U.S. population. We assumed nursing to be the only source of food for nursing infants, and calculation of their intake was based on the assumption that the daily consumption of human milk is 800 g (Dewey et al. 1991; Kent et al. 1999; Neville et al. 1988); milk PBDE levels were primarily levels previously reported (Schecter AJ, unpublished data; Schecter et al. 2003, 2005b). To calculate PBDE intake relative to body weight, we estimated an average weight of 7 kg for nursing infants [Centers for Disease Control and Prevention (CDC) 2000]. For other population groups, we obtained the median amount consumed each day for each of several categories of food from the U.S. Department of Agriculture (USDA) dietary intake survey (USDA 1999a, 1999b) and from Smiciklas-Wright et al. (2003). These intake estimates were multiplied by the mean PBDE concentration of our sample foods in each category to obtain a PBDE intake estimate for a “typical” man or woman (one whose food intake is at the 50th percentile) for each age group.

## Results

Tables 1–4 show the PBDE levels in U.S. food products reported on a whole weight basis. Of the 18 meat samples analyzed (Table 1), total PBDE levels varied from 39 ppt wet weight in a bacon sample to 1,426 ppt in one pork sausage sample. We observed considerable variation in levels, even between samples of the same type of meat, such as bacon, ground pork, and pork sausage. BDE-209 (deca-BDE), the major remaining congener in commercial production, was detected in 8 of the 18 meat samples and, when detected, ranged from 9.7 ppt in one ground beef sample to 245 ppt in ground turkey. Compared with PBDE levels in meat products in Spain (10–172 ppt) and Japan (6.25–63.6 ppt), PBDE levels were higher in the United States (Bocio et al. 2003; Ohta et al. 2002). Of the 24 fish samples analyzed (Table 2), total PBDE concentration ranged from 11 to 3,726 ppt. These values, although somewhat higher, are comparable with PBDE concentrations in fish of 88–1,019 ppt in Spain and 17.7–1,720 ppt in Japan (Bocio et al. 2003; Ohta et al. 2002). As with meat, we found considerable variation between samples of the same type of fish. Farm grown salmon and other fish tend to have higher PBDE concentrations than wild fish. Unfortunately, it is not always clear from the store label whether salmon or other fish were farm grown, because mislabeling appears to be common (Burros 2005). We found BDE-209 in 10 of the 24 fish samples, ranging from 4.9 ppt in a canned tuna sample to 1,269 ppt in a catfish sample. Of 15 dairy products analyzed (Table 3), the total PBDE concentration varied from 7.9 ppt in both whole and nonfat cows’ milk, to 683 ppt in cream cheese. We measured BDE-209 in 7 of the 15 dairy samples, with concentrations ranging from 9.1 ppt in lowfat yogurt to 481 ppt in cream cheese. Again, this was considerably higher than concentrations in these foods in Spain (10–48 ppt) (Bocio et al. 2003). Of the five types of miscellaneous food products analyzed (Table 4), eggs were lowest at 85 ppt and calf liver highest at 2,835 ppt total PBDEs. BDE-209 was found in all of the miscellaneous samples except margarine and varied from 10.3 ppt in eggs to 288 ppt in calf liver.

When PBDE concentrations are expressed on a lipid basis, fish still contain the highest levels, followed by meat and dairy products. Table 5 shows the PBDE levels for the various food types analyzed, on a lipid basis as well as on a wet weight basis. Although these lipid-normalized values reflect animal or fish body burdens of PBDEs, they are not useful in calculating dietary intake.

**Figure 1:**
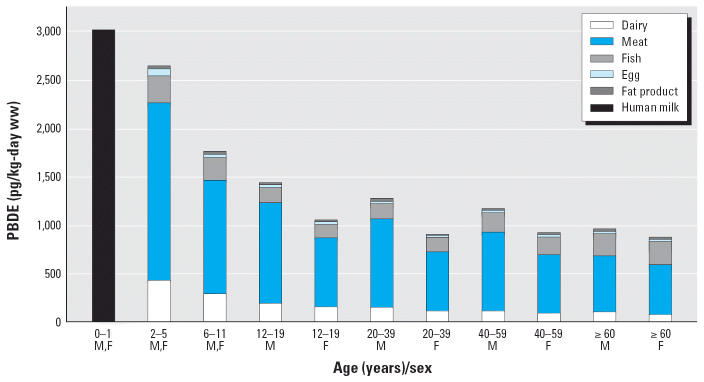
Daily PBDE dietary intake of U.S. population by age and food group (pg/kg body weight) as shown in Table 5. Abbreviations: F, female; M, male.

Table 6 and Figure 1 present estimates of dietary intake of PBDEs subdivided by food types for the U.S. population. In all groups > 1 year of age, total PBDE intake from meat is significantly higher than from any other food. As shown in Table 6, the highest dietary intake values of PBDEs are in nursing infants (307 ng/kg body weight per day, which compares to 1.0 or 0.9 ng/kg/day at ≥60 years of age for men and women, respectively; these are much higher than Swedish values of 0.63 and 0.58 ng/kg/day, respectively (Lind et al. 2002).

## Discussion

This larger U.S. market basket survey confirms that PBDE contamination levels in U.S. food are currently higher than previously reported in other countries (Bocio et al. 2003; Huwe and Larsen 2005; Ohta et al. 2002). Fish are highest in PBDE contamination on a whole weight basis, followed by dairy products and meat. Meat is the major source of PBDEs in the U.S. diet after nursing ends, followed by dairy products and fish, unlike some other countries where fish intake predominates (Bocio et al. 2003; Darnerud et al. 2001; Ohta et al. 2002). Men, with larger daily intakes of food, have a larger dietary intake of PBDEs than do women.

A large variation of PBDE levels exist, even for the same type of food (Huwe and Larsen 2005). Although the present study is the largest PBDE food survey in the United States to date to the best of our knowledge, we cannot claim that these new data are a representative sampling of the U.S. diet. Like other published surveys from other countries, the sample size needs to be increased and the samples need to be representative of the diet(s) of the country. Until this is done, uncertainty in estimates of food levels will exist, and as a result, intake estimates will be somewhat imprecise.

The comparatively higher PBDE levels in food cannot however be the only explanation for the 10- to 20-fold higher levels in blood and milk from the U.S. general population compared with European and Canadian levels (Bocio et al. 2003; Mazdai et al. 2003; Meironyte et al. 1999; Morland et al. 2005; Norén and Meironyté 2000; Ohta et al. 2002). Our total daily PBDE intake from dietary sources for adults is only 0.9–1.2 ng/kg body weight, which compares to Spain’s 1.2–1.4 ng/kg/day (Bocio et al 2003) and the United Kingdom’s approximately 1.5 ng/kg/day, assuming an average adult weight of 70 kg (Harrad et al. 2004), but is higher than Sweden’s 0.58–0.63 ng/kg/day (Lind et al. 2002). Although there is a great deal of uncertainty on half-lives of PBDEs, assuming a maximum half-life of 2 years (Geyer et al. 2004; Thuresson et al. 2006) and an American body composition of approximately 25% adipose tissue, PBDE intake from food would lead to a steady-state body burden of < 10 ppb lipid. Given that the median lipid-adjusted levels in the United States from recent blood, milk, and adipose specimens exceed 30 ppb lipid, and that those of the top 5% of the population are 10–100 times greater, it appears unlikely that diet is the sole or even major source of exposure to PBDEs. This is in direct contrast to the situation with PCBs and dioxins in which > 95% of the exposure of the general population comes from food (U.S. Environmental Protection Agency 2004). This suggests that other routes of intake might be more significant for PBDEs than is the case for dioxins and PCBs.

The trends in dietary intake of PBDEs show a decreased intake per kilogram of body weight with age, with the highest dietary intake during nursing in the first year of life, 307 ng/kg body weight. This is due to the high level of PBDEs in human milk (median, 1,056 pg/g wet weight), assuming that human milk was the only food consumed. Children 2–5 years of age have higher PBDE dietary intake per kilogram of body weight than do older persons because of higher food intake per kilogram of body weight.

PBDE congeners 47, 99, 100, 153, and 154, and in some cases 209, are major contributors in both food concentration and dietary intake estimates. This reflects the previously reported findings on the congener distribution in human blood (Schecter et al. 2005b).

As is true for dioxins and PCBs, human breast milk is a major source of daily exposure to PBDEs for infants. Based on lactational exposure to dioxins, the body burden of the infant does not exceed 3–5 times that of the mother, in spite of the 50–100 times greater daily intake (Abraham et al. 1996; Lorber and Phillips 2002). A similar situation may exist for PBDE exposure of nursing infants, in which case human milk can be a significant route of exposure for babies. We join Wu et al. (2005) in suggesting that, as well as food, routes of exposure such as house dust ingestion and inhalation are likely important pathways of PBDE intake for children as well as adults.

Extrapolating from rodent studies, MacDonald (2005) hypothesized that health risks are possible for more highly exposed persons in the U.S. general population. Although the health effects of the levels we report are not clear, it seems reasonable from a public health standpoint to reduce the levels of these chemicals in the environment.

## Figures and Tables

**Table 1:** PBDE levels (pg/g wet weight) in 18 U.S. meat samples.

Sample	Lipid (%)	BDE-17	BDE-28	BDE-47	BDE-66	BDE-77	BDE-85	BDE-99	BDE-100	BDE-138	BDE-153	BDE-154	BDE-183	BDE-209	Total PBDEs^a^
Bacon A	52.3	ND (5.2)	ND (7.1)	ND (78.8)	ND (5.2)	ND (5.2)	ND (5.2)	ND (28.8)	ND (6.8)	ND (5.2)	ND (5.2)	ND (5.2)	ND (5.2)	ND (166.6)	165
Bacon B	43.4	ND (0.4)	ND (2.1)	ND (19.9)	ND (0.4)	ND (0.2)	NA	ND (15.6)	ND (2.8)	ND (0.4)	ND (1.1)	ND (0.9)	ND (1.7)	ND (32.8)	39^b^
Bacon C	35.3	0.7	ND (2.0)	30.1	NA	NA	1.4	16.8	4.8	ND (0.7)	4.5	2.8	14.3	28.4	105^b^
Beef (ground) A	30.7	ND (3.1)	59.7	87.5	ND (3.1)	ND (3.1)	ND (3.1)	35.5	6.2	ND (3.1)	6.8	4.6	ND (4.2)	ND (95.7)	258
Beef (ground) B	13.6	0.2	ND (0.7)	23.4	0.5	NA	NA	32.3	4.5	0.4	4.7	2.5	NA	9.7	79^b^
Beef tenderloin	13.7	ND (1.4)	ND (1.5)	35.1	ND (1.4)	NA	1.7	40.3	6.9	ND (1.4)	4.9	3.7	3.8	ND (11.1)	105
Chicken breast	4.9	ND (0.04)	0.5	60.5	NA	NA	NA	128	17.1	2.2	12.0	10.8	3.2	48.5	283^b^
Duck	75.1	ND (0.5)	ND (3.0)	286	2.7	ND (0.3)	15.2	609	122	7.3	52.3	42.9	31.6	113	1,283^b^
Ground chicken	7.3	ND (0.7)	ND (1.5)	11.0	ND (0.7)	NA	ND (0.7)	18.9	4.6	ND (0.7)	4.1	2.6	5.8	80	129
Ground lamb	19.7	ND (2.0)	ND (2.1)	ND (23.0)	ND (2.0)	ND (2.0)	3.2	56.8	16.8	ND (2.0)	9.6	6.3	ND (2.0)	ND (150.6)	186
Ground pork	21.5	ND (2.2)	ND (3.5)	53.8	ND (2.2)	NA	3.1	74.2	12.9	4.3	18.7	15.0	19.9	ND (31.3)	221
Ground turkey	11.1	0.2	ND (0.5)	98	0.8	ND (0.1)	NA	217	54.4	3.9	32.9	24.1	36.8	245	713^b^
Pork	8.9	0.1	ND (0.5)	6.9	NA	NA	NA	16.3	1.8	0.2	1.0	1.2	1.3	11.7	41^b^
Pork sausage A	23.7	ND (1.3)	ND (6.9)	387	ND (1.0)	ND (0.3)	16.8	688	74.5	5.6	81.6	55.3	14.6	49.7	1,378^b^
Pork sausage B	24.4	ND (2.4)	ND (3.4)	39.4	ND (2.4)	ND (2.4)	2.6	71.6	8.3	ND (2.4)	22.0	13.7	10.7	ND (139)	244
Sausage A	26.2	ND (2.6)	ND (5.5)	ND (34.8)	ND (2.6)	NA	3.1	40.1	6.4	ND (2.6)	5.9	4.9	6.9	ND (51.0)	1,426
Sausage B	28.5	ND (2.9)	ND (3.2)	94.1	ND (3.5)	ND (2.9)	ND (2.9)	43.7	8.3	ND (2.9)	8.5	9.2	ND (2.9)	ND (41.7)	195
Wieners	32.9	ND (0.3)	ND (1.5)	386	1.4	ND (0.2)	11.1	703	53.9	7.2	106	49.8	14.3	ND (28.7)	1,348^b^
Mean	26.3	0.76	4.59	93.2	1.19	0.83	4.93	157	22.7	2.33	21.1	14	10.1	53.3	383
Median	24.1	0.66	1.03	39.4	1.08	0.57	2.62	42	7.57	1.37	7.68	5.63	5.83	38.1	190
Minimum	4.87	0.02	0.24	6.93	0.21	0.06	0.36	7.79	1.39	0.16	0.53	0.44	0.86	5.53	39
Maximum	75.1	2.62	59.7	387	2.74	2.62	16.8	703	121	7.28	106	55.3	36.8	245	1,426

Abbreviations: NA, not available; ND, not detected. LODs are shown in parentheses. Total PBDE levels and statistics for each congener were calculated by assuming that nondetected concentrations were one-half the LOD; for calculations, these were treated as zero.

a Totals rounded to the nearest whole number for hundreds and to the nearest decimal place for tens.

b Data from Schecter et al. (2004).

**Table 2:** PBDE levels (pg/g wet weight) of 24 U.S. fish samples.

Sample	Lipid (%)	BDE-17	BDE-28	BDE-47	BDE-66	BDE-77	BDE-85	BDE-99	BDE-100	BDE-138	BDE-153	BDE-154	BDE-183	BDE-209	Total PBDEs^a^
Canned tuna A	0.3	0.1	0.6	5.1	0.2	NA	0.2	3.2	0.6	ND (0.0)	0.3	0.2	1.1	4.9	16.6
Canned tuna B	0.5	ND (0.1)	0.2	2.1	0.2	NA	ND (0.1)	1.1	0.4	ND (0.1)	0.2	0.3	2.1	8.8	15.5
Catfish A	11.1	4.6	6.4	372	4.3	NA	NA	589	116	5.1	37.1	39.6	7.3	1269	2,450^b^
Catfish B	5.3	4.6	5.1	438	13.5	ND (0.1)	41.6	834	102	7.9	49.9	45.8	4.9	ND (15.9)	1,547^b^
Catfish C	5.2	2.2	3.7	137	0.7	ND (0.5)	11.7	184	39.5	ND (2.7)	15.8	15.2	ND (1.6)	ND (49.4)	437
Catfish fillet (farm)	5.7	1.1	3.7	197	6.3	NA	16.4	282	53.0	ND (4.1)	18.4	21.3	3.8	22.7	627
Halibut	0.2	0.6	4.1	76.6	2.8	NA	ND (0.1)	10.6	12.4	ND (0.1)	1.1	2.6	1.8	11.4	124
Herring	9.1	4.1	56.3	2,072	69.4	3.6	ND (0.9)	267	221	ND (0.9)	29.3	69.9	2.5	ND (26.4)	2,809
Mahi mahi	0.5	0.6	ND (2.0)	24.1	2.0	NA	0.6	13.0	5.1	ND (0.8)	1.4	4.9	4.3	ND (16.6)	66
Salmon A	8.0	79.2	92.6	1,222	30.6	ND (0.2)	NA	93.2	348	ND (0.2)	27.7	98.8	1.4	ND (9.0)	1,999^b^
Salmon B	13.9	118	142	2,081	59.1	ND (0.1)	NA	147	353	ND (0.2)	36.6	142	ND (1.2)	ND (7.0)	3,082^b^
Salmon C	10.3	18.4	49.4	1,103	35.3	ND (0.1)	ND (0.1)	239	217	ND (0.1)	18.3	45.1	ND (1.3)	ND (11.2)	1,732^b^
Salmon D	6.3	1.4	5.2	94.7	5.2	ND (0.9)	ND (0.6)	15.4	7.1	ND (0.6)	1.4	5.0	ND (0.8)	ND (9.1)	141
Salmon E	12.3	1.7	20.4	356	ND (2.1)	ND (1.2)	ND (1.2)	84.4	84.2	ND (1.2)	10.1	29.8	ND (1.4)	ND (29.2)	605
Salmon fillet (farm) A	7.4	11.1	50.5	1,000	63.1	NA	7.9	410	210	ND (1.4)	37.4	104	3.7	20.5	1,919
Salmon fillet (farm) B	6.9	2.3	27.9	517	24.3	NA	ND (0.7)	168	115	ND (0.7)	16.0	35.8	1.7	681	1,590
Sardines	9.6	3.3	53.6	2,748	85.6	ND (5.0)	ND (1.0)	358	257	ND (1.0)	51.9	139	ND (3.2)	ND (51.4)	3,726
Shark	0.4	1.1	29.8	784	29.5	0.3	NA	57.8	608	0.4	112	291	2.0	5.4	1,920^b^
Shrimp	0.6	0.3	3.6	75.6	NA	NA	NA	9.4	14.3	ND (0.1)	1.2	2.6	0.2	ND (1.3)	108^b^
Tilapia	1.0	ND (0.1)	ND (0.7)	5.9	NA	NA	0.1	1.3	0.6	ND (0.1)	0.2	0.5	ND (0.2)	ND (4.0)	11^b^
Trout A	4.2	4.8	22.2	320	NA	NA	ND (0.2)	79.8	66.5	0.2	11.8	26.3	4.4	ND (26.7)	549^b^
Trout B	10.1	4.3	49.3	826	ND (5.6)	ND (1.0)	ND (1.0)	128	198	ND (1.0)	24.7	61.3	2.5	ND (42.9)	1,319
Tuna	0.2	ND (0.1)	ND (1.0)	16.6	0.7	NA	ND (0.0)	ND (4.6)	2.9	ND (0.1)	ND (0.4)	ND (1.0)	0.5	23.4	48
Wild perch	1.2	ND (0.1)	0.7	10.2	0.4	NA	ND (0.1)	2.3	2.1	ND (0.1)	0.7	2.4	0.6	5.9	25
Mean	5.43	11.01	26.19	603	20.8	0.78	4.29	166	126	0.89	21	49.3	2.08	91.8	1,120
Median	5.52	1.97	5.77	338	5.23	0.30	0.35	88.8	75.3	0.33	15.9	28.	1.68	10.1	616
Minimum	0.15	0.03	0.20	2.11	0.18	0.06	0.02	1.15	0.43	0.02	0.21	0.21	0.12	0.63	11.14
Maximum	13.9	118	142	2,748	85.6	3.60	41.6	834	608	7.94	112	291	7.32	1,269	3,726

Abbreviations: NA, not available; ND, not detected. LODs are shown in parentheses. Total PBDE levels and statistics for each congener were calculated by assuming that nondetected concentrations were one-half the LOD; for calculations, these were treated as zero.

a Totals were rounded to the nearest whole number for hundreds and to the nearest decimal place for tens.

b Data from Schecter et al. (2004).

**Table 3:** PBDE levels (pg/g wet weight) of 15 U.S. dairy product samples.

Sample	Lipid (%)	BDE-17	BDE-28	BDE-47	BDE-66	BDE-77	BDE-85	BDE-99	BDE-100	BDE-138	BDE-153	BDE-154	BDE-183	BDE-209	Total PBDEs^a^
American cheese A	19.0	ND (1.9)	ND (1.9)	45.5	ND (1.9)	NA	ND (1.9)	34.9	5.91	ND (1.9)	3.67	2.67	2.09	14.6	114
American cheese B	11.6	ND (1.2)	ND (1.9)	28.2	0.80	NA	ND (1.2)	23.1	4.14	ND (1.2)	2.37	1.67	ND (1.2)	17.5	81.1
Gouda cheese	26.2	ND (2.6)	ND (3.0)	75.6	ND (2.6)	NA	5.52	57.4	12.2	ND (2.6)	8.27	4.76	1.6	ND (22.7)	182
Cottage cheese A	4.7	ND (0.5)	ND (1.5)	13.6	ND (0.5)	NA	ND (0.5)	14	2.63	ND (0.5)	1.39	1.01	ND (0.9)	ND (13.5)	41.5
Cottage cheese B	1.7	ND (0.2)	ND (1.3)	ND (6.9)	ND (0.2)	ND (0.2)	ND (0.2)	2.24	ND (0.4)	ND (0.2)	0.39	0.27	ND (0.3)	ND (4.2)	9.8
Cream cheese	39.2	0.4	ND (1.8)	97.8	1.57	ND (0.2)	NA	77.1	12.2	NA	5.96	2.84	ND (5.6)	481.4	683^b^
Milk (cow’s)	3.2	ND (0.3)	ND (0.3)	ND (5.6)	ND (0.5)	ND (0.3)	ND (0.3)	1.58	0.23	ND (0.32)	ND (0.3)	0.22	ND (0.3)	ND (3.5)	7.9
Evaporated milk A	6.6	ND (0.1)	ND (0.9)	15.8	NA	NA	NA	8.47	1.89	ND (0.2)	1.35	0.44	0.22	ND (1.9)	29.7^b^
Evaporated milk B	6.3	ND (0.1)	ND (0.9)	11.9	NA	NA	NA	12.6	2.27	0.20	1.91	0.80	ND (0.1)	ND (1.9)	31.1^b^
Goat milk	6.7	0.20	2.56	105	1.82	ND (0.07)	NA	97.9	27.3	NA	29	8.27	12.22	5.67	290^b^
Nonfat milk	0	ND (0.0)	ND (0.6)	ND (3.8)	ND (0.1)	NA	ND (0.1)	ND (2.5)	ND (0.8)	ND (0.1)	ND (0.1)	ND (0.2)	ND (0.1)	ND (7.5)	7.9^b^
Infant formula A	3.4	ND (0.0)	ND (0.5)	ND (3.1)	ND (0.1)	NA	NA	12.3	1.10	0.27	1.41	1.08	0.20	14	32.2^b^
Infant formula B	3.2	ND (0.1)	ND (1.2)	ND (7.7)	ND (0.1)	NA	NA	ND (5.1)	ND (1.5)	ND (0.3)	ND (0.5)	ND (0.3)	0.40	16.5	25.4
Lowfat yogurt	1.3	0.2	0.9	9.05	0.24	ND (0.02)	NA	7.78	1.39	0.05	0.97	0.40	1.44	9.08	31.6^b^
Ice cream	19.9	ND (0.2)	ND (0.8)	60.5	ND (1.0)	ND (0.4)	NA	63.7	9.45	ND (0.4)	6.91	3.41	5.47	ND (41.2)	171^b^
Mean	10.2	0.29	0.79	31.8	0.61	0.10	1.08	27.8	5.48	0.33	4.27	1.87	1.86	40.5	116
Median	6.3	0.16	0.63	13.6	0.24	0.10	0.24	12.6	2.27	0.20	1.41	1.01	0.45	9.08	32.2
Minimum	0.0	0.02	0.16	1.54	0.03	0.01	0.05	1.26	0.22	0.04	0.06	0.08	0.05	0.94	7.91
Max	39.2	1.31	2.56	105	1.82	0.20	5.52	97.9	27.3	1.31	29.0	8.27	12.2	481	683

Abbreviations: NA, not available; ND, not detected. LODs are shown in parentheses. Total PBDE levels and statistics for each congener were calculated by assuming that nondetected concentrations were one-half the LOD; for calculations, these were treated as zero.

a Totals were rounded to the nearest whole number for hundreds and to the nearest decimal place for tens.

b Data from Schecter et al. (2004).

**Table 4:** PBDE levels (pg/g wet weight) of five U.S. miscellaneous food samples.

Sample	Lipid (%)	BDE-17	BDE-28	BDE-47	BDE-66	BDE-77	BDE-85	BDE-99	BDE-100	BDE-138	BDE-153	BDE-154	BDE-183	BDE-209	Total PBDEs^a^
Chicken eggs (6)	11.5	0.14	0.20	22.5	0.24	0.03	1.52	36.6	5.93	0.58	3.63	2.56	0.68	10.32	85^b^
Butter	78.3	0.4	1.3	165	1.2	NA	NA	172	40.4	4.5	16.8	12.6	5.3	66.2	485^b^
Calf liver	6.4	0.1	0.4	9.0	NA	ND (0.6)	0.5	10.5	1.6	ND (0.2)	2.9	1.8	6.2	81.6	115^b^
Chicken liver	6.4	0.3	1.1	687	5.3	NA	27.2	1,258	261	17.9	148	130	11.5	288	2,835^b^
Margarine	83.3	ND (0.7)	ND (2)	ND (12)	ND (2.3)	ND (1)	ND (1.1)	ND (7.2)	ND (2)	ND (1.4)	0.9	ND (0.9)	ND (2.6)	ND (142)	88^b^

Abbreviations: NA, not available; ND, not detected. LODs are shown in parentheses. Total PBDE levels and statistics for each congener were calculated by assuming that nondetected concentrations were one-half the LOD; for calculations, these were treated as zero.

a Totals were rounded to the nearest whole number for hundreds and to the nearest decimal place for tens.

b Data from Schecter et al. (2004).

**Table 5:** PBDE concentrations (pg/g wet weight) in the survey items.

		Lipid-based				Wet weight/whole weight			
	No. of samples	Minimum	Mean	Median	Maximum	Minimum	Mean	Median	Maximum
Human milk^a^	62	6,000	66,000	32,000	419,000	31	1,916	968	21,359
Meat									
Poultry	4	1,708	3,919	3,771	6,423	129	602	498	1,283
Beef	3	581	729	766	840	79	147	105	258
Pork	2	461	744	744	1,028	41	131	131	221
Bacon	3	90	234	298	316	39	103	105	165
Processed meat	5	684	3,408	4,097	5,814	195	918	1,348	1,426
Dairy									
Ice cream	2	859	1,645	1,645	2,431	31.6	101.3	101.3	171
Milk	2	NA	NA	NA	NA	7.9	7.9	7.9	7.9
Cheese	6	577	866	697	883	9.8	185	97.6	683
Eggs	1	NA	739	739	NA	NA	85	85	NA
Fat									
Margarine	1	NA	106	106	NA	NA	88	88	NA
Butter	1	NA	619	619	NA	NA	485	485	NA
Fish	24	1,100	37,319	17,408	480,000	11	1,119	616	3,726

NA, not available.

a Data from Schecter AJ (unpublished data) and Schecter et al. (2005b).

**Table 6:** Daily PBDE dietary intake from food sources (pg/kg, or parts per quadrillion bw).

	Age (years)/sex/bw										
Food	0–1 M and F (5 kg)	2–5 M and F (16 kg)	6–11 M and F (29 kg)	12–19 M (55 kg)	12–19 F (49 kg)	20–39 M (70 kg)	20–39 F (60 kg)	40–59 M (70 kg)	40–59 F (60 kg)	≥60 M (70 kg)	≥60 F (60 kg)
Dairy											
Ice cream and ice milk	0	63	59	33	35	25	17	27	20	30	22
Milk	0	191	113	61	42	29	27	29	25	30	28
Total cheese	0	173	121	118	83	90	62	56	46	37	28
Total dairy		427	293	212	160	144	106	112	91	97	78
Meat											
Poultry	0	790	477	449	344	413	331	396	331	275	291
Beef	0	211	177	187	126	168	93	134	88	95	74
Pork	0	52	36	37	23	34	22	34	22	32	22
Bacon	0	6	4	4	2	3	2	3	3	4	3
Processed meat	0	780	476	334	215	300	164	244	153	178	120
Total meat		1,839	1,170	1,011	710	918	612	811	597	584	510
Fish											
Total fish	0	280	232	163	137	160	149	208	187	224	243
Eggs											
Total eggs	0	69	38	31	24	27	21	30	23	32	26
Fat											
Margarine	0	6	6	3	4	3	3	4	4	5	4
Butter	0	30	17	9	10	14	8	7	8	14	8
Total fat products		36	23	12	14	17	11	11	12	19	12
Human milk	306,560	0	0	0	0	0	0	0	0	0	0
Sum PBDE intake per body weight (pg/kg-day ww)	306,560	2,652	1,755	1,429	1,045	1,264	900	1,172	912	957	869

Abbreviations: bw, body weight; F, female; M, male; ww, wet weight.
